# Study on Accuracy Improvement of Slope Failure Region Detection Using Mask R-CNN with Augmentation Method

**DOI:** 10.3390/s22176412

**Published:** 2022-08-25

**Authors:** Shiori Kubo, Tatsuro Yamane, Pang-jo Chun

**Affiliations:** 1 Institute of Industrial Science, The University of Tokyo, Chiba 277-8574, Japan; 2 Department of International Studies, The University of Tokyo, Chiba 277-8561, Japan; 3 Department of Civil Engineering, The University of Tokyo, Tokyo 113-8656, Japan

**Keywords:** image augmentation, Mask R-CNN, slope failure, image segmentation, deep learning

## Abstract

We proposed an automatic detection method of slope failure regions using a semantic segmentation method called Mask R-CNN based on a deep learning algorithm to improve the efficiency of damage assessment in the event of slope failure disaster. There is limited research on detecting landslides by deep learning, and the lack of training data is an important issue to be resolved, as aerial photographs are not taken with sufficient frequency during a disaster. This study attempts to use CutMix-based augmentation to improve detection accuracy. We also compare the detection results obtained by augmentation of multiple patterns. In the comparison of the not augmented data case, the recall increased by 0.186 in the case using the augmented data with the shape of the slope failure region maintained. When the image data was augmented while maintaining the shape of the slope failure region, the recall score indicated the low oversights in the prediction result is 0.701. This is an increase of 0.186 compared to the case where no augmentation was performed. In addition, the F1 score was 0.740, this also increased by 0.139, and high values were obtained for other indicators. Therefore, the method proposed in this study is greatly useful for grasping slope failure regions because of the detection with high accuracy, as described above.

## 1. Introduction

As shown in landslide inventory maps [1,2], in recent years, sediment disasters such as slope failures and debris flows caused by earthquakes and heavy rainfalls due to global warming have been occurring in many parts of the world and causing extensive damage. In August 2009, Typhoon Morakot hit Taiwan, causing massive slope failure, mudslides, and flooding [3]. This caused many houses in the downstream villages to be buried under soil, and 400 of the approximately 450 villagers were buried in a tragic situation. Additionally, in Uttarakhand, India, heavy rains in June 2013 caused slope failures that affected 1800 villages, killing 4120 people, collapsing 15 bridges, and causing other serious damage [4]. In Hokkaido, Japan, a massive Mj 6.7 earthquake in 2018, which occurred the day after Typhoon Jebi arrived in Hokkaido, caused more than 5600 slope failures [1]. As a result, many homes were destroyed, and roads were blocked. The earthquake claimed the lives of 41 people, and 36 people died from landslides [5]. In addition, during the heavy rains in the northern part of Kyushu, located in the western part of Japan, record-breaking rainfall within a small area caused multiple slope failures. It brought about severe damage to both Fukuoka and Oita Prefectures in Japan. There were 34 fatalities in Fukuoka prefecture, and a large number of these, a ratio of 56%, were caused by landslides [6]. In July of the following year, concentrated heavy rains also occurred in the western parts of Japan, and large-scale debris flow and slope failures occurred from heavy rains. Especially in Hiroshima prefecture, there were many landslides and 87 people, or approximately 80% of the, dead from these landslides [7]. As landslides, slope failures, debris flows caused by earthquakes, and concentrated heavy rains occur in multiple areas simultaneously, it is necessary to understand the situation over a broad area. Additionally, it is difficult to assess the situation on-site immediately after a disaster strikes because roads may be cut off by sediment. For these reasons, aerial photographs are often used to grasp the disaster situation without visiting the damaged area. However, the interpretation of data is conducted manually by engineers, which takes much time. Of the disasters reported from 1988 to 2017, landslides and mass displacements affected approximately 5 million people. The current rapid urbanization and population growth in landslide-prone areas are causing large-scale human suffering [1]. Considering the urbanization in many areas of the country in the future, it is necessary to discuss the creation of information for rapid response during disaster recovery and relied on efforts.

There are many methods for detecting regions damaged by disasters, such as Afaq et al. [8], who showed the algebra, artificial intelligence, and GIS-based techniques. In the method of using AI, many studies have been conducted to grasp the damage situation quickly at the time of the slope failures using a wide range of images and video, including aerial photographs captured from a disaster relief helicopter, optical and synthetic aperture radar (SAR) images captured from artificial satellites, and video captured using unmanned aerial vehicles (UAV). Aimaiti et al. created a landslide map showing the places landslides occur using the coherence, slope gradient, and SAR image intensity difference threshold, which are calculated by SAR images captured in the descending and ascending orbits before and after the disaster, Digital Elevation Model data [9]. In addition, Miura et al. estimated the changing area after the earthquake (i.e., the slope damage region) by calculating and comparing the Normalized Difference Vegetation Index (NDVI), based on optical images captured before and after the disaster [10].

As described above, there is much research in which damage regions are estimated by comparing images captured before and after the disaster. However, there are some concerns, such as the fact that there may not necessarily exist images captured before the disaster; even if the images exist, they may be unclear due to the weather conditions, and the topography may be different before the disaster, and at the time the image was taken due to changes in the use of the land. For these reasons, it is desirable to estimate slope failure regions using machine learning/deep learning without using images before the disaster. For instance, Amit et al. estimated semantic segmentation of the areas of damaged regions such as slope failures and flooding from newly captured images containing the damaged area to use the convolutional neural network (CNN) model, which trained the features of the damaged area by the aerial photographs capturing the disaster regions in the past [11]. Furthermore, Kawamura et al. detected the sediment movement regions using a CNN model, which was trained with grayscale aerial photographs of the regions where sediment movement sections exist and do not exist [12]. Ghorbanzadeh et al. calculated NDVI values, slope, and plan curvature. From optical satellite images and used them to detect landslide locations using various methods [13]. In addition, Ghorbanzadeh et al. used a UAV to photograph mountainous terrain regions that included slope failure regions and created a trained model [14].

In recent years, high-resolution satellite images and aerial photographs have become available within a short time after a disaster. An increasing number of studies have been conducted to detect damaged areas using only post-disaster data [15]. There are several concerns with the recently studied methods for assessing damage, such as the large number of data required compared to using only post-disaster images, which may require more time to prepare all the data, and the possibility that pre-disaster images may not exist for the disaster location. Based on the above, in this study, we detected slope failure regions through deep learning using aerial photographs taken during past disasters instead of pre-disaster photographs of the disaster location to rapidly and automatically grasp situations when slope failures occurred due to disasters, such as heavy rain and earthquakes, among others. At this time, we performed slope failure detection using Mask R-CNN, which is generally known as an instance segmentation method, as a semantic segmentation method. The goal is to create a detection model to estimate the size of disasters and distribution in the future. An instance segmentation method that detects and classifies candidate object regions at the pixel level is employed as this method can grasp the number of slope failure regions and the distribution. In this study, we first attempted to detect slope failure regions from small images cropped to a specific size to verify whether it is possible to detect them with high accuracy. As a future task, we are also considering the number and distribution of slope failure regions from images taken over a wide area, so it is desirable to adopt this method, which can determine not only the area (i.e., number of pixels) of the detection area, but also the number of detected objects and their distribution (i.e., coordinates).

In addition, the images used to assess damage can only be taken at the time of a disaster, making insufficient training data an issue in research on damage assessment using such images. Ghorbanzadeh et al. used the freely available Sentinel-2 data and ALOS digital elevation model, rather than high resolution satellite images to detect landslide regions using the FCN algorithm and further evaluate the generalizability and transferability of the constructed models [16]. In addition, they a performed deep learning model with rule-based object-based image analysis (OBIA) for landslide areas with distinct features, rather than a pixel-based deep learning approach [17]. In the case of limited training datasets, for example, Oh et al. developed a patch-based convolutional neural network approach with a relatively small number of trainable parameters to analyze the chest X-rays (CXR) for the diagnosis of COVID-19, a globally prevalent disease [18]. In addition, Shahabi et al. developed an unsupervised learning model by employing a convolutional auto-encoder (CAE), taking into account the limited number of data available for training [19]. In order to improve the loss of detection accuracy caused by this issue, we attempted to improve the detection accuracy of the slope failure region using augmentation. When using deep learning to detect slope failure regions, the multiple sets of data that the Geospatial Information Authority of Japan provides are used. Based on several previous studies [12,13,20], it is estimated that thousands to tens of thousands of satellite images are required for accurate detection. For this reason, augmentation is performed on the captured images in many studies, including these. There are various methods of augmentation, such as Cutout [21], Mixup [22], and CutMix [23].

This study aims to grasp the effect of the training data augmented by the CutMix method that stitches together multiple images with image labels to create a new image on the detection accuracy. Then, the slope failure region is detected by a learning model trained on the image data augmented. In the infrastructure field, the authors’ group has also applied machine learning, including deep learning, to detect concrete clacks and grooves [24,25,26], asphalt clacks [27,28], corrosion of steel girders [29,30], and evaluation of durability estimation of corroded steel [31,32], and description of bridge damage [33,34] has been utilized and useful results have been obtained. However, it would be useful to show the augmentation impact on disaster cases quantitatively. In this study, the multiple training data augmentation method is considered to detect the slope failure regions with high accuracy using such little data. Based on the above, this study aims to rapidly and automatically detect slope failure regions using Mask R-CNN, employing post-disaster aerial photographs as training data created using the augmentation method. The topography and land use are not mentioned, but rather the area where slope failures occur in various locations, such as bare land and covered with forest vegetation, are detected, and the characteristics of the areas where failure regions are difficult to detect are discussed.

## 2. Detection Model of Slope Failure Regions

### 2.1. Image Recognition Method

#### 2.1.1. Slope Failure Monitoring

Image segmentation categorizes input images at the pixel level, and a common method is a semantic segmentation. Semantic segmentation is a method used for performing class categorization concerning all pixels in the image. It is used for detecting cracks in concrete bridges and road surfaces [35,36]. In addition, as a development of the same, it can estimate candidate regions by an object and perform instance segmentation by classifying the type of object at the pixel level. Panoptic Segmentation combines these techniques. In contrast to semantic segmentation, which performs class categorization concerning all pixels, instance segmentation performs detection and categorizing at the pixel level about the object candidate region, making it a method that grasps the form of the target object with greater accuracy. As mentioned above, in consideration of the future grasping the scale of disaster such as the distribution and area size, Mask R-CNN, which is generally known as an instance segmentation method is used for semantic segmentation to segmentation slope failure regions in this study.

#### 2.1.2. Mask R-CNN

As a successor to the object detection method R-CNN (Regions with CNN features) [37], which was proposed in 2014, Mask R-CNN (Mask Region with convolution Neural Network) [38] was proposed. Mask R-CNN adopts the instance segmentation described above; it is a multi-task method that performs semantic segmentation in addition to object detection, which is used in R-CNN. R-CNN is a two-stage type method that performs class categorizing concerning the respective estimated candidate regions while performing object region detection from the input image. This makes the processing complex, and there is the issue that it requires much time. Following this, development is progressing on Fast R-CNN [39] that can perform end-to-end training for tasks other than estimating the candidate region, and Faster R-CNN [40] that can perform end-to-end training for all tasks. Training speed and object detection accuracy are gradually improving. Then, Mask R-CNN was released in 2017. Capturing feature values for each candidate region has improved and added a new segmentation task.

**Figure 1:**
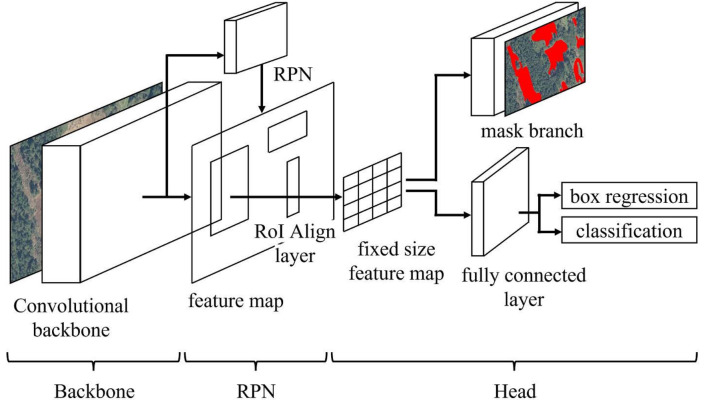
Mask R-CNN framework.

Figure 1 shows a schematic diagram of the Mask R-CNN structure. Mask R-CNN is comprised of three parts: Backbone that extracts features from the input image, RPN (Region Proposal Network) that selects candidate regions within the image, and Head that estimates and identifies feature values for each candidate region from the RoI pooled feature map and performs estimation and semantic segmentation of the detailed position of the object. In Faster R-CNN, the predecessor to Mask R-CNN, in the Head section, RoI pooling was used when selecting features for each candidate region from the feature map. RoI pooling is a simple type of pooling in which the spatial pyramid pooling [41] pyramid structure is excluded, and the processing contains the candidate region within a fixed-size feature vector. This method has the issue that it is impossible to accurately estimate the mask by discretizing the feature values at a fixed resolution. Therefore, Mask R-CNN adopts RoI Align, which estimates without losing the feature map information. With RoI Align, sampling values are calculated from the surrounding four pixels of each cell in the candidate region. Pooling the results creates a fixed-size RoI feature vector. In this way, by considering sub-pixel-level information, it is possible to eliminate misalignments at RoI pooling. In Mask R-CNN, as visualization is performed at the pixel level of the detected region through semantic segmentation, it is important to accurately estimate the boundary section of the candidate region, which has a significant impact on accuracy. For this reason, the mask accuracy of the object region in Mask R-CNN greatly increases using RoI Align as described above [38]. Mask R-CNN employs cross-entropy as the loss function, which is the sum of three loss terms: a loss term from a class categorizing and regressing a bounding box, and a term for estimating the semantic segmentation mask. This defined as a multitask loss function as shown in Equation (1). The same weights are applied to all these loss functions in this research to calculate the total loss. The first and second terms on the right-hand side are defined by Equation (2).

(1) $$Loss=L_{CLS}+L_{REG}+L_{MASK}$$

(2) $$L_{CLS}+L_{REG}=\frac{1}{N_{cls}}\sum_{i}L_{cls}\left(p_{i},p_{i}^{*}\right)+\lambda \frac{1}{N_{reg}}\sum_{i}p_{i}^{*}L_{reg}\left(t_{i},t_{i}^{*}\right)$$

which is similar to that of the Faster R-CNN described above [40]. where, *i*, $p_{i}$, $t_{i}$, $p_{i}^{*}$, $L_{cls}$, $t_{i}^{*}$, and $L_{reg}$, are, respectively, the index of the anchor in the mini-batch, the predicted probability that anchor i contains an object, the rectangular region indicating the predicted object position by vector values, the binary label data, the loss on class categorizing in the two classes, the rectangular region of the correct, and the regression loss on the prediction of the rectangular region which is denoted by $L_{r}eg\left(t_{i},t_{i}^{*}\right)=R\left(t_{i}-t_{i}^{*}\right)$ with robust loss *R*. Furthermore, $N_{cls}$ and $N_{reg}$ are the size of the mini-batch and the number of anchors, respectively. λ is a hyperparameter to keep balance the loss of class categorizing and regression, and in this study, λ=10 as the same value in [38]. The third term on the right-hand side of Equation (1) is the average value of the binary cross-entropy for an arbitrary number of class labels. The network can generate masks for all classes without causing interference between classes by defining this term.

### 2.2. Datasets

**Figure 2:**
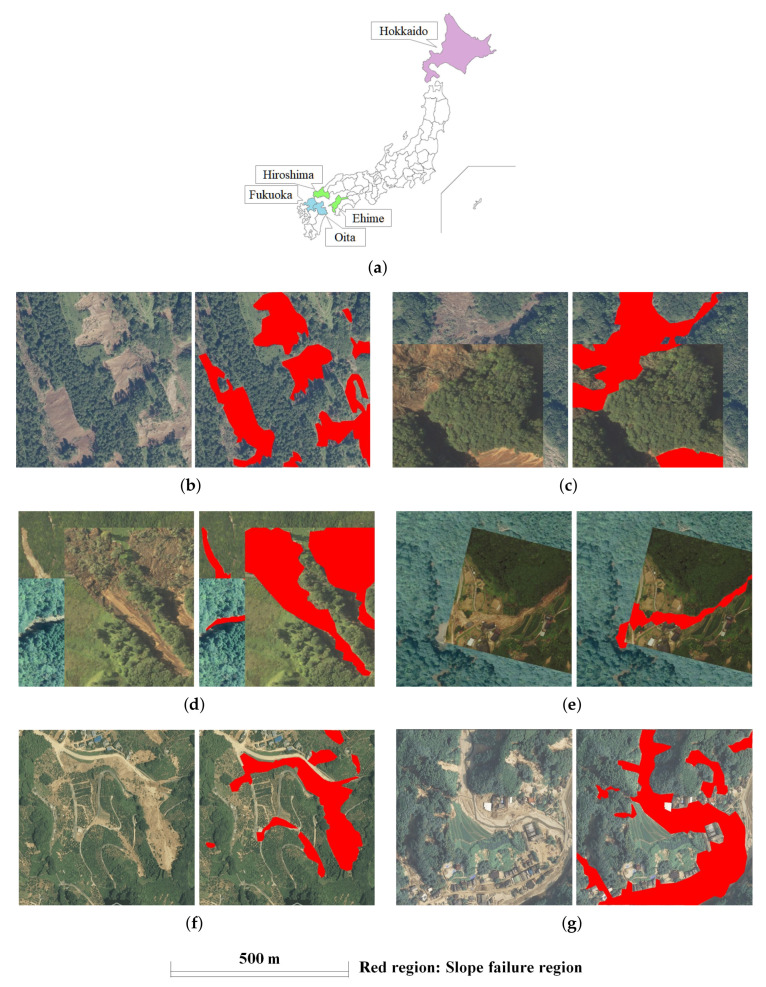
Images used in each case (**Left**: Aerial photograph. **Right**: Ground truth.) (**a**) Aerial photograph acquisition areas (The blue areas are the areas affected by the July and September 2017 heavy rains; aerial photographs of these areas were used as test data. The green and purple areas are the areas affected by the July 2018 heavy rains and earthquake, respectively; aerial photographs of these areas were used as training and validation data.) (**b**) Original image used in Case 1. (**c**) Image with one time CutMix processing used in Case 2. (**d**) Image with two times CutMix processing used in Case 3. (**e**) Image with rotation and warping applied to the one time CutMix image used in Case 4. (**f**) Image for validation case. (**g**) Image for test case.

The data for this study was compiled from slope failures that occurred in Fukuoka and Oita Prefectures during Kyushu’s heavy rains in July 2017, slope failures that occurred in Ehime and Hiroshima Prefectures due to heavy rains in July 2018, and slope failures that occurred in Hokkaido in September 2018 as a result of the Hokkaido Eastern Iburi earthquake. In this study, training images, including slope failure regions, were created by capturing slope failure regions from aerial photographs [42] taken after the disaster, published by the Geospatial Information Authority (GSI). An aerial camera took the images used in this study. The aerial camera is equipped with a GNSS receiver called GNSS/IMU device. IMU to measure the camera position and attitude during shooting can capture high-precision images. These images can be viewed and downloaded from a web map site called GSI Map provided by the Geographical Survey Institute (https://maps.gsi.go.jp/ (accessed on 26 July 2022)). As shown in Figure 2, for each image showing a slope failure area, some images show only one slope failure, and some show multiple slope failures. Many of the images were clearly identified as slope failures, but for those that were not, we used images identified as slope failures by the Geospatial Information Authority of Japan (GSI) or by us during disaster surveys. The size of all captured images is 1024 px × 1024 px vertically and horizontally, and the resolution is 0.5 m/px for all images. For training and validation data, 788 images were created from aerial photographs captured during the 2018 west Japan heavy rain disaster and the Hokkaido Eastern Iburi Earthquake in September 2018 (the green and purple areas in Figure 2a). The images created were then divided at a ratio of 4:1, giving 591 images as training data and 197 images as validation data. In addition, based on aerial photographs captured at the time of the heavy rains in Northern Kyushu in July 2017, 145 images were created and used as test data (The blue areas in Figure 2a). Test data’s size and shooting procedure are the same as training and validation data. Then, all of the data used for verifying and testing each model was the same. In order to develop a general algorithm for slope failure phenomena, this study does not distinguish between slope failures caused by different factors, such as earthquakes and heavy rainfall.

### 2.3. Image Augmentation

As mentioned above, few aerial photographs of slope failure areas are taken during disasters. Most studies that use deep learning to detect slope failure areas improve the detection accuracy by augmentation. In this study, we created the four patterns of training data shown in Table 1 and trained each model constructed using the various hyperparameters and optimization methods shown below to grasp the effect of the method of each augmentation method on the validation and testing results.

For Case 1, we used only the 591 training data images described in the previous section (Figure 2b). We did not perform augmentation (referred on Case 1 in Table 1). Next, Cases 2, 3, and 4 are used 12,411 images for training. However, different augmentation methods are used for each (referred on the training data of Cases 2, 3, and 4 in Table 1). For Case 2, we took the images after performing CutMix once on the 591 original images and created 20 images for each original image. By creating 11,820 images augmented as shown in Figure 2c, we trained using 12,411 images. CutMix is a method of augmentation in which a section of multiple images is cut off and reconnected to make one image. With CutMix, as the training data is connected with labels, this is a method of augmentation. It is possible to prevent a decrease in training efficiency. This provides it a higher level of accuracy than similar methods, such as Mixup and Cutout [23].

For Case 3, CutMix performed in Case 2 is performed twice, and the images shown in Figure 2f are created in the same number as for Case 2. For Case 4, rotation and warping processing is performed at a fixed probability for the cut images after CutMix. The images shown in Figure 2g are created in the same number as Case 2. Increasing the diversity of training data and regularization effect are promised by stitching together multiple images with labels to create a new mixed image, such as CutMix. It is difficult to distinguish the bared area as slope failure regions by color because the colors of these areas are similar. We use CutMix to focus on the shape features of slope failure regions as the detection target is a certain regularity in the shape area, such as a slope failure region. It is believed to differentiate between slope failure regions and bared areas.

### 2.4. Model Construction and Training

#### 2.4.1. Construction of Semantic Segmentation Model

In the model used in this study, ResNet101 is used for the backbone, showing the feature value section of the network. ResNet101 is used to perform training based on the difference after subtracting the input from the output rather than looking for the optimal output obtained from a specific layer to solve the issues such as the vanishing gradient problem and degradation problem caused by the depth of the network. As described above, the all-input image size is standardized at 1024 px × 1024 px. The learning rate, optimization method, and weight decay are 0.001, SGD (Stochastic Gradient Descent), and 0.0001, respectively. These parameters are determined following in He et al. [38]. In this study, Google Colaboratory is used, and then, the GPU is NVIDIA Tesla P-100 (about 16 GB). Therefore, considering the memory size used in this study, the batch size is set to 3, considering the memory size used in this study. Additionally, the weight that has already been trained using the MS COCO dataset as a pre-training weight is employed.

#### 2.4.2. Cnn Training and Validation

**Figure 3:**
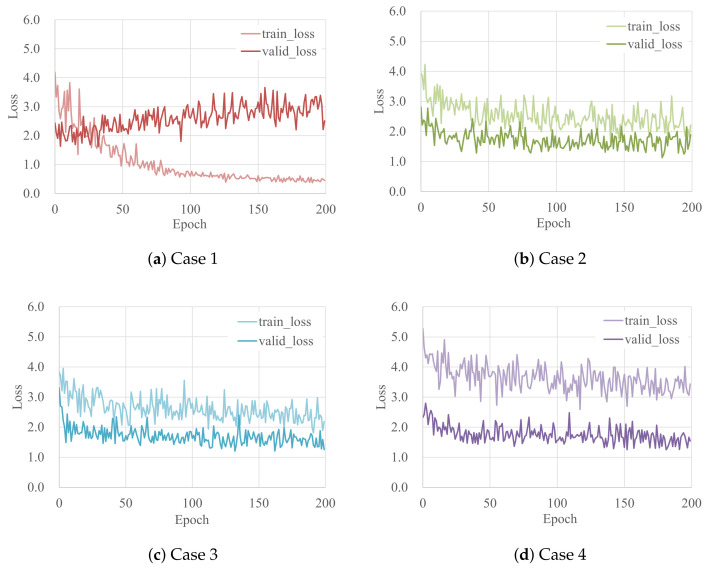
Training and validation loss in each case.

These detection models were trained using four sets of training data described above. Figure 3 depicts the transition in the loss during training and validation for each case. As described above, Mask R-CNN employs cross-entropy as the loss function, which is the sum of three loss terms: a loss term from a class categorizing and regressing a bounding box, and a term for estimating the semantic segmentation mask. According to Figure 3a, in Case 1, where no augmentation was performed, the loss during training gradually decreased, and the smallest value occurred during the 190th training interval, at 0.374. On the other hand, the loss gradually increased at validation. As the data used for training was smaller, overtraining of the data occurred. In Cases 2, 3, and 4, the number of training data items was increased. This stabilized for both training and validation and was seen to decrease gradually. In addition, comparing Case 2 (Figure 3b), in which CutMix was performed once, with Case 3 (Figure 3c), in which it was performed twice, it is found that there are approximately the same values for both training and validation, so the number of times CutMix is performed was not seen to have a significant impact on loss for each.

Furthermore, although the learning loss in Case 4 is larger than in Cases 2 and 3, the validation loss is not much different from Cases 2 and 3 (Figure 3d). In all three cases except Case 1, the training loss always exceeds validation loss. That is to say, it is possible that this model cannot learn the training data efficiently as the training data becomes complex by augmentation such as CutMix. The unprocessed validation data has continuity in the slope failure regions. However, the training data is augmented by CutMix, but lacks continuity compared with the original and validation data. Therefore, the latter loss is assumed to be higher than the former. In this study, among the training results for each model, the test was performed using the weight with the smallest loss step when performing the validation shown in Table 2.

## 3. Results of Detection

### 3.1. Results of Slope Failure Detection

**Figure 4:**
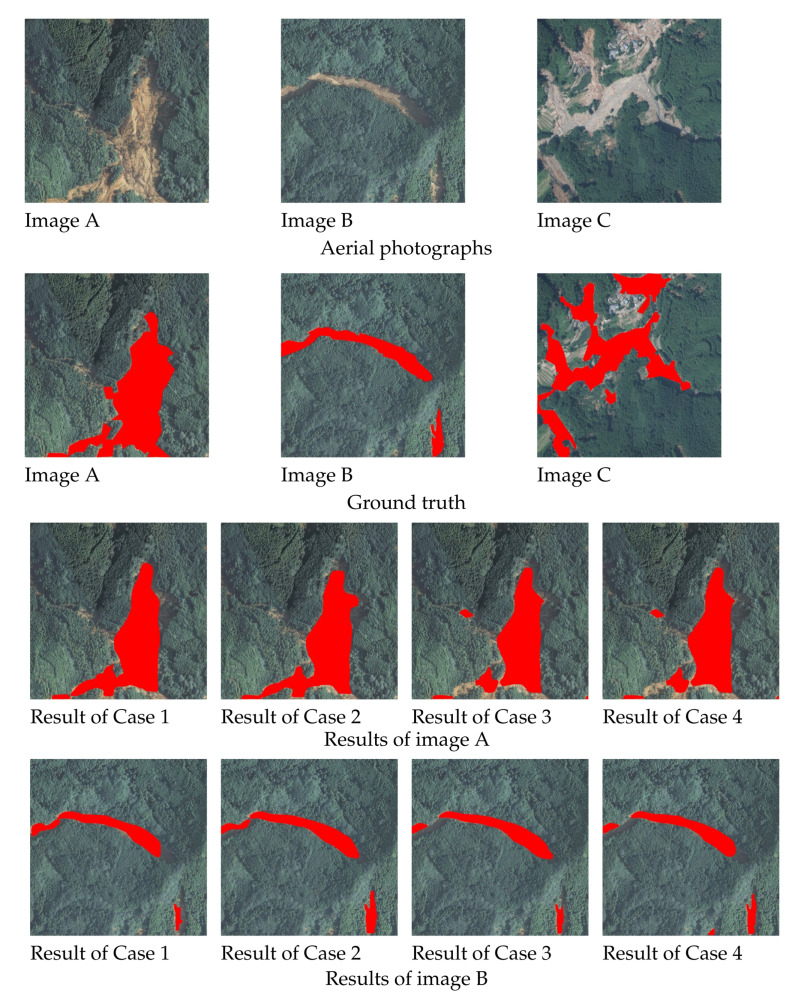

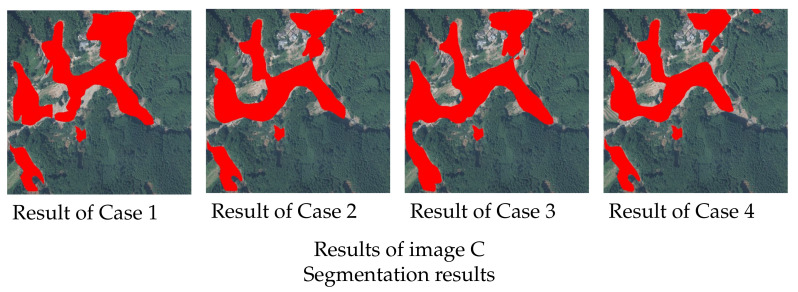
Segmentation results (Examples of detectable cases). The images in first and second lines show the original aerial photographs and the ground truth, which indicates the area of slope failure regions in red in the aerial photograph, respectively. The images in the third to fifth lines show the results of detecting slope failure regions for images A, B, and C shown in the first line, respectively. The detection results are shown in the order of Case 1, 2, 3, and 4 from the left to right. Even if the slope failure region has a complex shape, the slope failure regions can be recognized in all detection results.

**Figure 5:**
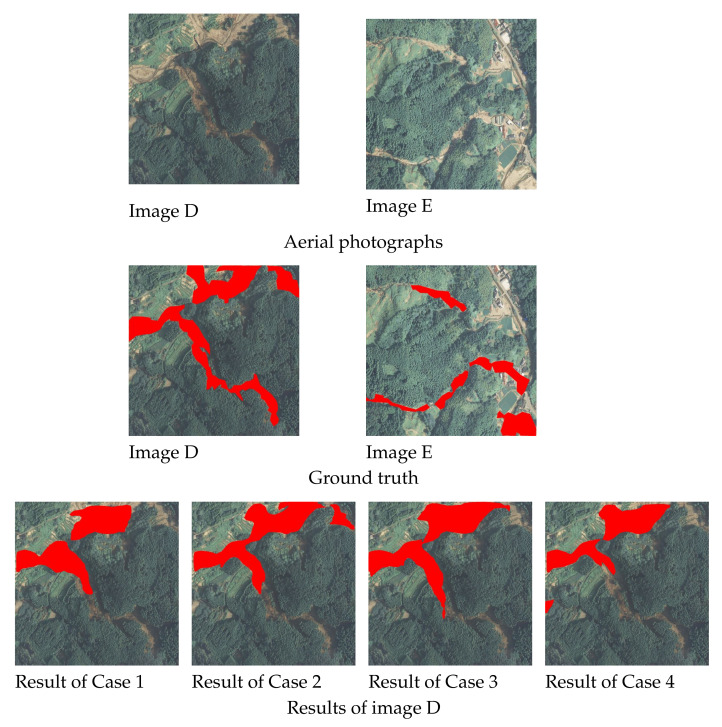

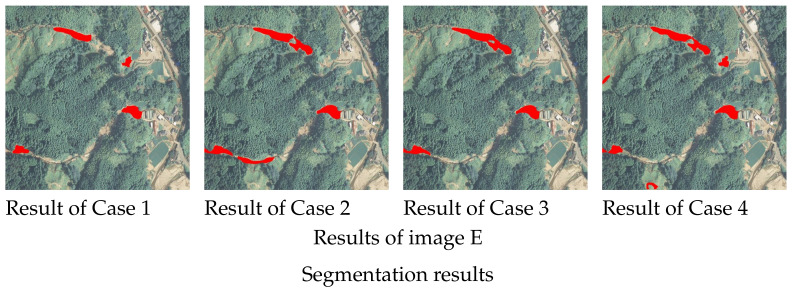
Segmentation results (Examples of partially undetectable cases). The images in first and second lines show the original aerial photographs, and the ground truth, which indicates the area of slope failure regions in red in the aerial photograph, respectively. The images in third to fourth line show the results of detecting slope failure regions for images D and E shown in the first line, respectively. The detection results are shown in the order of Case 1, 2, 3, and 4 from the left to right. Where the failure region is exceptionally detailed and is blocked due to being surrounded by trees or shadows such that it cannot be detected, even using human eyes, it may be difficult to judge.

**Figure 6:**
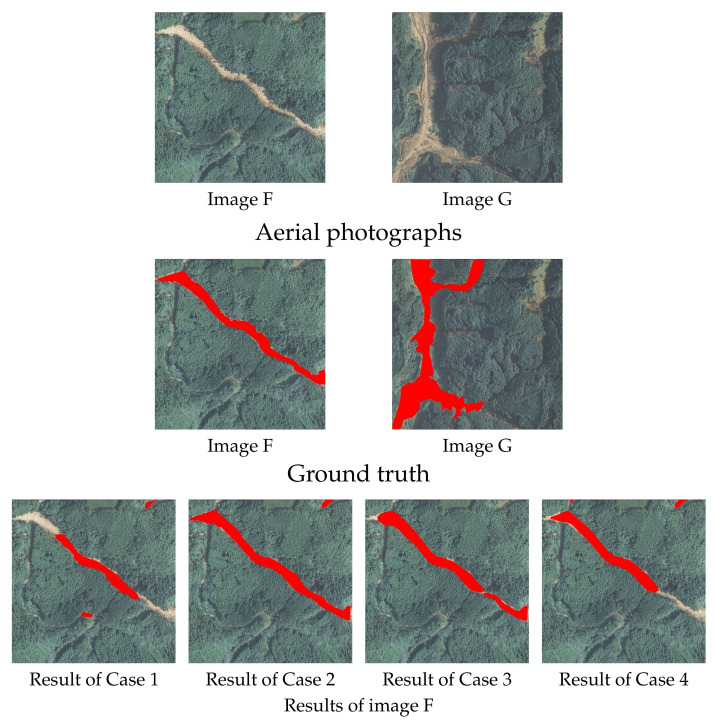

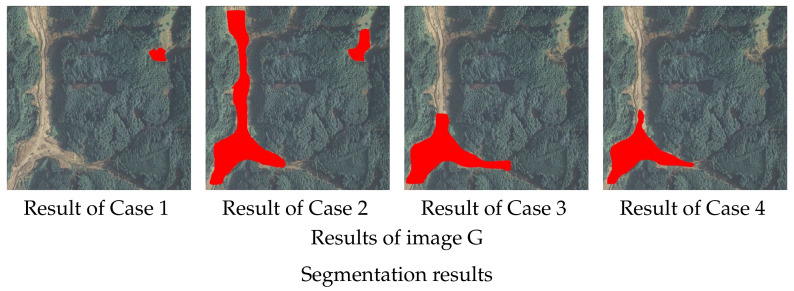
Segmentation results (Examples of Cases 2, 3, 4, and 1 detected correctly in that order). The images in the first and second lines show the original aerial photographs and the ground truth, which indicates the area of slope failure regions in red in the aerial photograph, respectively. The images in the third to fourth lines show the results of detecting slope failure regions for images F and G shown in the first line, respectively. The detection results are shown in the order of Case 1, 2, 3, and 4 from the left to right. Case 2 is generally detected correctly in most cases, while the other cases have more detection omissions in the order of case 3, 4, and 1.

The regions of slope failures were detected using the trained model constructed in the previous section. In all cases described above, 145 images are used for test data. However, a few examples are shown in this paper due to space limitations. Examples of detection results that were detected correctly for all cases are shown in Figure 4, examples in which they were not detected correctly, in any case, are shown in Figure 5, and examples where the regions that could be detected accurately decreased in the order of Cases 2, 3, 4, 1, are shown in Figure 6. In each figure and the detection results, the original and correct images are shown in the top section of the figure.

From Image A (Figure 4), the shapes of slope failure regions can be recognized in all of the detection results in the situation the slope failure occurs over a wide area. Other than Case 4, it is possible to capture complex shapes at the bottom of the image. Moreover, in the case of Image B (Figure 4), even when the shape of the failure region is particularly detailed, it can largely be detected in all cases. In addition, misdetection of the non-failure region did not occur in any of the cases. Only the failure regions were appropriately detected, even for images, including non-damaged areas, such as houses and roads, as shown in Image C (Figure 4). Hence, it can be judged that regardless of the number of data items used in the training and method of augmentation, if the collapse area is visible, slope failure regions are detectable in all cases. As mentioned above, there are many cases where slope failure regions could be detected in all cases. However, some cases could be difficult to detect. However, there were no situations where slope failure regions within the image could not be detected for all cases. More than half of the cases consisted of areas where they could be at least partially detected. As shown in Image D (Figure 5), in cases involving damage over a wide area, as seen at the top of the image, this could be effectively detected.

These areas cannot be detected in many cases if the failure region is surrounded by trees or in a shadow, as shown at the bottom. Similarly, these are detected in many images, such as Image B in Figure 4 in cases where the failure regions are extremely detailed, but not in some images, such as Image E in Figure 5, they were only partially detected. For cases like Image E in Figure 5, where the failure region is exceptionally detailed and is blocked due to being surrounded by trees or shadows such that it cannot be detected, even using human eyes, it may be difficult to judge whether these are non-failure regions, such as rivers or roads, or failure regions including debris. As shown above, the surrounding trees and the tree shadows make detecting the slope failure region difficult. Among the slope failure regions that the detection model could not detect, there were cases where even humans could not accurately detect whether the area was a slope failure region, a road, or a river. As described above, there were cases where the collapse area was not detected when it was very narrow or when trees or their shadows hid it. However, in Cases 2 and 3, the entire regions were detected correctly in most cases, as shown in Figure 4. As shown in Figure 5, it is mostly Cases 1 and 4 that are not detected correctly. For example, in Image F and G, as shown in Figure 6, the regions for which the failure region cannot be detected correctly tend to decrease in the order of Cases 2, 3, 4, and 1. As shown in Image F (Figure 6), in Cases 2 and 3, both edges of the failure region can be accurately detected. On the contrary, in Case 1, neither side of the failure region, and in Case 4, the bottom of the image one-third cannot be detected. As for Image G (Figure 6), only a part of the failure region in the upper part of the image was not detected in Case 2, except the lower part of the failure region vertically in the image was not detected in Cases 3 and 4, and the entire area was not detected in Case 1. Hence, although Case 2 is generally detected correctly in many cases, the other cases, Cases 3, 4, and 1, have more detection omissions in that order.

### 3.2. Accuracy Assessment

Based on all detection results, the accuracy of each model is evaluated. The accuracy of each model can be evaluated based on the detection results from each section. In this study, we perform the calculations following equations using TP, FP, FN, and TN as shown in the confusion matrix (Table 3). Each model was evaluated regarding the accuracy, precision, recall, specificity, and F1 score. Accuracy is the ratio at which failure regions and non-failure regions are correctly detected in terms of the whole image; precision is the ratio that failure regions were correctly detected as failure regions; recall is the ratio of the places where failure regions are correctly detected as failure regions; specificity is the ratio that non-failure regions were correctly detected as non-failure reasons, and the F1 score is the harmonic mean of the precision and recall rates described above.

(3) $$\mathrm{Accuracy}=\frac{\mathrm{TP}+\mathrm{TN}}{\mathrm{TP}+\mathrm{FN}+\mathrm{FP}+\mathrm{TN}}$$

(4) $$\mathrm{Precision}=\frac{\mathrm{TP}}{\mathrm{TP}+\mathrm{FP}}$$

(5) $$\mathrm{Recall}=\frac{\mathrm{TP}}{\mathrm{FP}+\mathrm{TN}}$$

(6) $$\mathrm{Specificity}=\frac{\mathrm{TN}}{\mathrm{FP}+\mathrm{TN}}$$

(7) $$F1\mathrm{score}=\frac{2(\mathrm{Precision}\times \mathrm{Recall})}{\mathrm{Precision}+\mathrm{Recall}}=\frac{\mathrm{TP}}{1+\frac{\mathrm{FN}+\mathrm{FP}}{\mathrm{TP}}}$$

**Figure 7:**
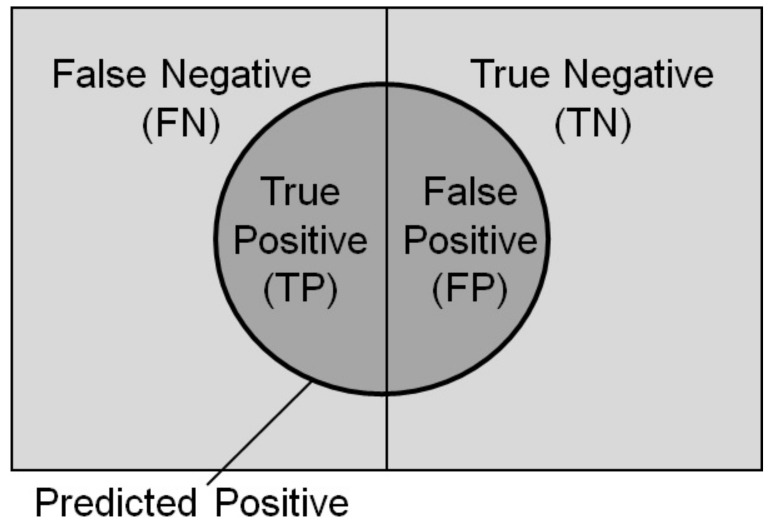
Confusion matrix overview.

A confusion matrix created based on the detection results for each case is shown in Table 4. Then, the sum of pixels in the detection area (i.e., red area in the result figures) in all test images is shown in Table 4. The confusion matrix is shown in Figure 7. The red regions in the test images are TP and FP, indicated by circles in Figure 7. Each pixel is sorted according to the confusion matrix (Table 3), respectively. In this study, the non-slope failure region is everything except slope failure, as only the detection of the slope failure region is conducted in Mask R-CNN. That is, subtracting the number of pixels in the slope failure region from the total number of pixels in a single image yields the number of pixels in the non-slope failure region. The detection accuracy calculated based on the confusion matrix is shown in Table 5. Based on Table 4, pixels (TP, TN) where the failure regions/non-failure regions were correctly detected as failure/non-failure regions are most common in Case 2. On the contrary, pixels (FN) where the failure regions were most frequently erroneously detected as non-failure regions were most common in Case 1, and pixels (FP) where non-failure regions were most erroneously detected as failure regions were most common in Case 3. From the calculation results (Table 5) for each detection accuracy calculated based on these results, we can see that Case 2 had the highest values for the three indicators of accuracy, recall, and the F1 score. There was no significant difference in indicators other than recall or the F1 score between Cases 1 and 4, where limited training data and overtraining occurred. The low recall was the cause of the low F1 score. The recall was the lowest in all cases, implying that many actual failure regions were missed and misidentified as non-failure regions. Comparing Cases 2 and 3, Case 3 had the lowest value for all five indicators, even though the number of trained image data was the same as Case 2. It is attributed to performing CutMix twice when augmenting the training data. As a result, it is assumed that the training data became more complex, and information on the form of the damaged region was lost. This means it had the highest ratio of correctly detecting failure regions and correctly detecting non-failure regions as non-failure regions.

Furthermore, when comparing the precision of Case 1 and Case 3, the difference was only 0.009. When comparing the recall of Case 1 and Case 4, the difference was only 0.026. As a result, it can be concluded that increasing the training data does not always improve detection accuracy, as shown in Cases 3 and 4, even when the training data is increased, based on the fact that even when training data is augmented, this is at approximately the same accuracy as cases like Case 1, where there is less training data, and that when compared with Cases 2 and 3, Case 2 has higher values for indicators, and to increase the detection accuracy, it is considered preferable to not perform processing in a way that greatly changes the form of the target region including the original image when augmenting the training data. The previous section stated that the recall of each case decreased in the order of Cases 2, 3, 4, and 1. Using the evaluation metrics, it is found that Case 2 has the highest detection accuracy. As the training model created in this study is envisaged to grasp the situation at the time of the disaster, it is expected that the number of oversights in detecting disaster occurrence areas should be reduced as much as possible. For this reason, recall, which shows the ratio of actual slope failures correctly detected as slope failure, is considered the most important indicator. In addition, as it is also important to detect failure regions accurately, it is better to consider the F1 score. For this reason, when looking at the training models constructed in this study, the most useful for grasping the situation at the time of the disaster were the two training models with the highest recall and F1 score.

Each model was evaluated in terms of accuracy, precision, recall, specificity, and the F1 score. Accuracy is the ratio at which failure regions and non-failure regions are correctly detected in terms of the whole image; precision is the ratio that failure regions were correctly detected as failure regions; recall is the ratio of the places that were failure regions being correctly detected as failure regions; specificity is the ratio that non-failure regions were correctly detected as non-failure reasons; and the F1 score is the harmonic mean of the precision and recall rates described above.

A confusion matrix created based on the detection results for each case is shown in Table 4. Then, the sum of pixels in the detection area (red area in the result figures) in all test images are shown in Table 4, and each pixel is sorted according to the confusion matrix (Table 3), respectively. In this study, non-slope failure region is everything except slope failure region since only the detection of slope failure region is conducted in Mask R-CNN. The detection accuracy calculated based on the confusion matrix is shown in Table 5. Based on Table 4, pixels (TP, TN) where the failure regions/non-failure regions were correctly detected as failure/non-failure regions are most common in Case 2. On the contrary, pixels (FN) where the failure regions were most frequently erroneously detected as non-failure regions were most common in Case 1, and pixels (FP) where non-failure regions were most erroneously detected as failure regions were most common in Case 3. From the calculation results (Table 5) for each detection accuracy calculated based on these results, we can see that Case 2 had the highest values for the three indicators of accuracy, recall, and the F1 score. There was no significant difference in indicators other than recall or the F1 score between Cases 1 and 2, where training data was limited and overtraining occurred.

The low recall was the cause of the low F1 score. The recall was the lowest in all cases, implying that a large proportion of actual failure regions were missed and misidentified as non-failure regions. Comparing cases 2 and 3, Case 3 had the lowest value for all five indicators, even though the number of trained image data was the same as Case 2. It is attributed to perform CutMix twice when augmenting the training data. As a result, it is assumed that the training data become more complex and information on the form of the damaged region was lost. This means it had the highest ratio of correctly detecting failure regions and a high ratio of correctly detecting non-failure regions as non-failure regions. Furthermore, when comparing the precision of Case 1 and Case 3, the difference was only 0.009, and when comparing the recall of Case 1 and Case 4, the difference was only 0.026. As a result, it can be concluded that increasing the training data does not always improve detection accuracy. As shown in Cases 3 and 4, even when the training data is increased, based on the fact that even when training data is augmented, this is at approximately the same accuracy as cases like Case 1, where there is less training data, and that when compared to Cases 2 and 3, Case 2 has higher values for indicators, and to increase the detection accuracy, it is considered preferable to not perform processing in a way that greatly changes the form of the target region including the original image when augmenting the training data. In the previous section, it was stated that the recall of each case decreased in the order of Cases 2, 3, 4, and 1. Using the evaluation metrics, it is found that Case 2 have the highest accuracy in detection.

As the training model created in this study is envisaged to be used to grasp the situation at the time of disaster, it is expected that it will reduce failures in detection areas where disasters have occurred. For this reason, recall, which shows the ratio of actual slope failures correctly detected as slope failure, is considered to be the most important indicator. In addition, as it is also important to detect failure regions accurately, it is better to also consider the F1 score. For this reason, when looking at the training models constructed in this study, the most useful for grasping the situation at the time of the disaster was considered to be the two training models with the highest recall and F1 score.

## 4. Discussion

In this study, automatic detection of slope failure regions by Mask R-CNN uses only images of the damaged regions to create the information to expedite recovery activities after the disaster. In some studies, slope failure images and pre-disaster information such as geographical information and pre-disaster image of the target area have been used as input data such as Ref. A and B [11,14]. In order to clarify the position of this study, in addition to these studies, previous studies used only slope failure region images as input data, such as Ref. C and D [12,43], which are also described, in comparison to our study. As this study’s images and augmentation method differs from previous studies, only the comparison is provided for reference purposes.

The detection accuracies of Case 1 without augmentation, Case 2 with the most accurate in this study, and four previous studies are shown in Table 6. Then, if multiple cases have been examined in previous studies, the case with the best accuracy is described for Ref. A and B, not only slope failure images, but also other information such as geographical information, the pre-disaster image of the target area, and an ortho-mosaic map generated from slope failure images have been used as input data. For Ref. C and D, only the images of past disasters are used as training data, and post-disaster images are used as validation and test data. The authors conduct Ref. D, and the detection methods and parameters are similar to this study. Table 6 shows that the case of using the combination of multiple data in addition to post-disaster images such as Ref. A and B obtain higher accuracy than using only slope failure regions images such as Ref. C and D. This depends on the method adopted for the training model and the images. However, the most significant factor is the variety of data used. Although the accuracy obtained using the proposed method is worse than that of Ref. A and B, which use multiple data, the method of Case 2 obtained higher accuracy than the Ref. C and D used only images taken at the damaged area. Case 1, in which no augmentation is performed, has the same accuracy as Ref. D where augmentation is performed by rotating the training data. However, augmentation with CutMix, etc., as shown in Case 2 resulted in higher recall and F1 score than Ref. D. The method employed in this study enabled a significant reduction in the number of oversights. Detecting the damaged regions at disaster occurred by machine learning model trained using only the post-image of disaster leads to provide information to disaster site requiring urgent action quickly. On the other hand, it is better to use as in Ref. A and B if multiple data are available, as the detailed and accurate information on the disaster site is needed at some time after the occurring disaster. The difference between this study and previous studies (Ref. A and B) is the variety of data. In addition, the differences between this study and previous studies (Ref. C and D) that used post-disaster images for training are that grayscale images are used as training data (Ref. C), the augmentation is not performed (Ref. C), and that only rotation is used as the augmentation method (Ref. D). Therefore, it can be said that the influence of the color of aerial photographs and the use of arbitrary augmentation methods to training data contributed greatly to the improvement of accuracy. We considered that it is important to have a large number and variety of data since A and B, which employed multiple data as training data, were more accurate than this study. Therefore, as a measure for improvement, it may be effective to employ new images of narrow slope failure regions or areas darkened by tree shadows as training data, or to construct a new detection model specifically for such regions, which were not detected in this study.

## 5. Conclusions

In order to quickly and accurately assess the damaged situation when slope failures occur due to disasters such as heavy rain or earthquakes, the slope failure region was detected using Mask R-CNN. In this study, the effects of image augmentation on detection accuracy were grasped. The results showed that augmentation with CutMix increased recall by 0.186 and F1 score by 0.139. In addition, the accuracy of other indicators were equal to or higher than that of the case where augmentation is not performed. The detection model trained using augmentation data created by maintaining the shape of the failure region was more accurate in detecting the failure region than trained using augmentation data created with complex processing that would significantly degrade the shape of the failure region shown in the original image. In addition, comparing cases of using training data in which complex augmentation processing had been performed multiple times on the original image and cases in which only the original image was used as training data without performing augmentation, similar values were obtained for each evaluation matrix. In other words, the effect of increasing the amount of data on accuracy is small when complex processing is performed on training data. It is important to consider the purpose for which it is to be used since the criterion for what constitutes high accuracy are vague. For example, practitioners think that it is fast and accurate enough in terms of the disaster assessment in the initial stage, which is assumed to be the unambiguous purpose. In this study, only post-disaster images are used as training data for detecting slope failure regions. In order to obtain higher accuracy with such as restriction on the type of training data, it is necessary to use methods not investigated such as new augmentation focusing on these undetected regions and adjusting the image’s brightness or contrast. This study similarly treats slope failure regions caused by different factors such as earthquakes and heavy rainfall. However, it is possible to capture the feature of the collapse point for each factor, which may lead to improved accuracy.

## Figures and Tables

**Table 1:** Breakdown of images used.

	Training Data		Validation Data	Test Data
	Images	Augmentation		
Case 1	591 images	No	197 images	145 images
Case 2	12,411 images	One time cutmix		
Case 3		Two times cutmix		
Case 4		One time cutmix, rotation, warping		

**Table 2:** Minimum validation loss.

	Value/Number of Epoch
Case 1	1.621/ 32
Case 2	1.129/178
Case 3	1.200/132
Case 4	1.255/152

**Table 3:** Confusion matrix.

	True Class	Slope Failure Regions	Non-Slope Failure Regions
Prediction Class			
Slope failure regions		TP (True Positive)	FP (False Positive)
Non-slope failure regions		FN (False Negative)	TN (True Negative)

**Table 4:** Breakdown of detection result in each case.

	True Class	Slope Failure Regions	Non-Slope Failure Regions
Prediction Class			
Slope failure regions	Case 1	6,527,912	2,518,387
	Case 2	8,891,079	2,447,233
	Case 3	8,650,994	3,175,846
	Case 4	6,865,760	1,603,504
Non-slope failure regions	Case 1	6,153,051	136,844,170
	Case 2	3,789,884	136,915,324
	Case 3	4,029,969	136,186,711
	Case 4	5,815,203	137,759,053

**Table 5:** Accuracy of detection results.

	Case 1	Case 2	Case 3	Case 4
Accuracy	0.943	0.959	0.953	0.951
Precision	0.722	0.784	0.731	0.811
Recall	0.515	0.701	0.682	0.541
Specificity	0.982	0.982	0.977	0.988
F1 score	0.601	0.740	0.706	0.649

**Table 6:** Comparison of detection accuracy.

	Case 1 of This Study	Case 2 of This Study	Ref. A [11]	Ref. B [14]	Ref. C [12]	Ref. D [43]
Accuracy	0.943	0.959	-	-	-	0.949
Precision	0.722	0.784	0.93	0.89	0.214	0.741
Recall	0.515	0.701	0.94	0.82	0.784	0.578
Specificity	0.982	0.982	-	-	-	0.982
F1 score	0.601	0.740	0.93	0.85	0.336	0.650

## Data Availability

Not applicable.
